# Polysaccharide utilization loci from Bacteroidota encode CE15 enzymes with possible roles in cleaving pectin-lignin bonds

**DOI:** 10.1128/aem.01768-23

**Published:** 2024-01-05

**Authors:** Andrea Seveso, Scott Mazurkewich, Sanchari Banerjee, Jens-Christian Navarro Poulsen, Leila Lo Leggio, Johan Larsbrink

**Affiliations:** 1 Wallenberg Wood Science Center, Division of Industrial Biotechnology, Department of Life Sciences, Chalmers University of Technology, Gothenburg, Sweden; 2 Department of Chemistry, University of Copenhagen, Copenhagen, Denmark; University of Milano-Bicocca, Milan, Italy

**Keywords:** carbohydrate esterase, carbohydrate esterase family 15, glucuronoyl esterase, lignocellulose, pectin, protein structure

## Abstract

Lignocellulose is a renewable but complex material exhibiting high recalcitrance to enzymatic hydrolysis, which is attributed, in part, to the presence of covalent linkages between lignin and polysaccharides in the plant cell wall. Glucuronoyl esterases from carbohydrate esterase family 15 (CE15) have been proposed as an aid in reducing this recalcitrance by cleaving ester bonds found between lignin and glucuronoxylan. In the Bacteroidota phylum, some species organize genes related to carbohydrate metabolism in polysaccharide utilization loci (PULs) which encode all necessary proteins to bind, deconstruct, and respond to a target glycan. Bioinformatic analyses identified CE15 members in some PULs that appear to not target the expected glucuronoxylan. Here, five CE15 members from such PULs were investigated with the aim of gaining insights on their biological roles. The selected targets were characterized using glucuronoyl esterase model substrates and with a new synthetic molecule mimicking a putative ester linkage between pectin and lignin. The CE15 enzyme from *Phocaeicola vulgatus* was structurally determined by X-ray crystallography both with and without carbohydrate ligands with galacturonate binding in a distinct conformation than that of glucuronate. We further explored whether these CE15 enzymes could act akin to pectin methylesterases on pectin-rich biomass but did not find evidence to support the proposed activity. Based on the evidence gathered, the CE15 enzymes in the PULs expected to degrade pectin could be involved in cleavage of uronic acid esters in rhamnogalacturonans.

**IMPORTANCE:**

The plant cell wall is a highly complex matrix, and while most of its polymers interact non-covalently, there are also covalent bonds between lignin and carbohydrates. Bonds between xylan and lignin are known, such as the glucuronoyl ester bonds that are cleavable by CE15 enzymes. Our work here indicates that enzymes from CE15 may also have other activities, as we have discovered enzymes in PULs proposed to target other polysaccharides, including pectin. Our study represents the first investigation of such enzymes. Our first hypothesis that the enzymes would act as pectin methylesterases was shown to be false, and we instead propose that they may cleave other esters on complex pectins such as rhamnogalacturonan II. The work presents both the characterization of five novel enzymes and can also provide indirect information about the components of the cell wall itself, which is a highly challenging material to chemically analyze in fine detail.

## INTRODUCTION

The complex architecture of biomass, as well as its recalcitrance to enzymatic deconstruction, represents a significant challenge to lignocellulose-based biorefineries (1, 2). Recalcitrance is a multifactorial property (3), but a feature recognized to make a significant contribution is the presence of covalent bonds between lignin and hemicellulose polysaccharides, the so-called lignin-carbohydrate complexes (LCCs) (4–6). Lignin is primarily a component of the secondary cell wall and is present in only minor amounts in the primary cell wall (7). LCC covalent linkages include ester and ether bonds, as well as glycosidic linkages, which cross-link the heterogeneous lignin polymer to carbohydrates in the plant cell wall material (4–6). Glucuronoyl esterases (GEs) are enzymes that have been shown to cleave LCC ester bonds (8–10) and are classified into carbohydrate esterase family 15 (CE15) within the Carbohydrate-Active enZymes Database [CAZy; http://www.cazy.org (11)]. Since their discovery, the principal activity reported of this family is GE, cleaving esters of d-glucuronic acid (GlcA, often 4-*O*-methylated) moieties (10, 12), and with the proposed role of decoupling lignin and glucuronoxylan. While enzymes of both fungal and bacterial origins have been discovered and characterized, bacterial CE15 enzymes have shown a greater variety in sequence and structure and, in some cases, a certain promiscuity on model substrates (10), raising questions regarding their main biological role. 4-*O*-Methylation has been shown to be an important activity determinant for fungal GEs (13, 14) but has not been investigated in detail for bacterial enzymes. In contrast to fungal GEs (13, 15), several bacterial CE15 members were recently found to be active on esters of galacturonic acid (GalA) (16), possibly indicating a different, and yet undefined, role for some family members (2).

Pectin is a heterogenous polysaccharide rich in d-GalA found in the plant cell wall which contributes to structural strength and integrity. Its composition can vary largely, depending on plant type, ranging from 2% to 35% of the total dry biomass (17), and it can comprise a significant proportion of biofuel feedstocks such as sugar beet pulp (~20% dry weight) (18). Pectin is found primarily in primary cell walls and in the middle lamella between cells, and only in smaller amounts in secondary walls (19). In nature, pectin occurs as homogalacturonan (HG) and rhamnogalacturonans (RG-I and RG-II) with HG being the major constituent [up to 60% (20)]. HG is composed of long chains of α-(1,4)-linked d-galacturonate, which can be methyl esterified and acetylated at the C2 or C3 positions. Despite lignin and pectin being enriched in different cell wall regions, earlier studies have suggested that pectin could be covalently attached to lignin within the cell wall or middle lamella (19, 21), i.e., as part of LCCs, and more recently, further features were observed, indicating possible cross-links of pectin with hemicelluloses and phenolic compound such as those found in lignin (22, 23). Moreover, deeper studies on LCCs have allowed obtainment of fractions proposed to contain pectin and lignin (24), and recently, a study inferred the direct ester linkage between HG and lignin from studies on wild type and mutants of switchgrass and *in vitro* produced lignin-pectin co-polymers (25). However, enzymes with activity on such complexes have not been identified.

Members of the Bacteroidota phylum (previously Bacteroidetes) can be found in diverse environments such as soil, marine, or fresh water, and many recent studies have focused on their role as dominant members of animal guts (26, 27). The phylum includes many species recognized as highly proficient degraders of complex carbohydrates, which can be viewed as a great source of carbohydrate-active enzymes (CAZymes). Their polysaccharide degradation efficiency is often linked to their genomic organization of CAZyme-encoding genes into so-called polysaccharide utilization loci (PULs) (26). Typical Bacteroidota PULs cluster all genes encoding CAZymes, as well as carbohydrate-binding proteins, transporters, and regulators that enable deconstruction and response to the specific polysaccharide the PUL targets. The archetypical PUL is the starch utilization system (Sus) in *Bacteroides thetaiotaomicron*, which has been extensively studied since its discovery (28). The Sus uses a cell surface-bound amylase (SusG), surface-bound starch-binding proteins, and a maltooligosaccharide-binding transport complex (SusC/D) that enables import of generated oligosaccharides into the periplasm. Once in the periplasm, sequestered from competing organisms, oligosaccharides are further degraded to glucose by the glucosidases SusA and SusB. PUL proteins are believed to be expressed at a low basal level until the target polysaccharide is sensed, which in the Sus is governed by SusR, which upon binding to maltooligosaccharides upregulates the entire Sus. Other PULs are expected to follow the same principles, where sensing of a target glycan specifically upregulates the PUL genes (29), and PULs targeting a wide range of polysaccharides have been characterized in recent years (26, 30–34), including highly complex RGII (34). Homologs of the tandem *susC/D* gene pair are signatures of Bacteroidota PULs and now used to identify putative ones from genomic sequences (35). Identifying predicted PULs and characterizing novel encoded enzymes from them can be a successful strategy to discover new activities or modules (34, 36–38), and their importance to the field was highlighted with the addition of the Polysaccharide-Utilization Loci DataBase (PULDB) in CAZy in 2018 (35). Xylan-targeting PULs have been discovered previously (39), with some containing CE15 enzymes (35, 40) which could be expected based on the ability of GEs to cleave bonds between glucuronoxylan and lignin. There are also CE15 members encoded in PULs which appear to target non-xylan polysaccharides such as pectin, suggesting novel roles for these putative enzymes.

The aim of the present study was to investigate putative CE15 enzymes from uncharacterized PULs which do not have clear connection to xylan degradation to gain more insight into their biological roles. We mined the PULDB for putative non-xylan targeting PULs encoding CE15 enzymes and chose five representative cases from the bacteria *Alistipes shahii*, *Bacteroides intestinalis*, *Parapedobacter indicus*, *Phocaeicola dorei* (formerly *Bacteroides dorei*), and *Phocaeicola vulgatus* (formerly *Bacteroides vulgatus*), all of which reside in animal guts except *P. indicus*, which was isolated from soil (41). Of these, *B. intestinalis*, *P. dorei*, and *P. vulgatus* have been shown to grow on a range of polysaccharides including xylan (42–44), though for the remaining two species, extensive studies have not been performed. All the CE15 targets investigated functioned as efficient GEs on canonical uronic acid ester substrates, with two of them having very high catalytic efficiency. Comparative analysis with a new GalA-based model substrate, proposed to mimic pectin-LCC, was used for the first time to study GEs and, additionally, whether the CE15 enzymes could act as pectin methylesterases (PMEs) or in synergy with other pectin-degrading enzymes was explored.

## MATERIALS AND METHODS

### Molecular biology and protein production

CE15 genes were amplified by PCR from genomic DNA, using the primers shown in Table S1, from *Alistipes shahii* WAL 8301 DSM 19121, *Bacteroides intestinalis* DSM 17393, *Parapedobacter indicus* DSM 28470, *Phocaeicola dorei* DSM 17855, and *Phocaeicola vulgatus* ATCC 8482. The amplified products were cloned into pET-28a vectors containing an N-terminal His_6_ tag and were subsequently transformed into *Escherichia coli* HST08 Stellar cells (Clontech). Constructs were confirmed by DNA sequencing (Eurofins) and subsequently transformed into *E. coli* BL21 (λDE3) for protein production. Transformed cells were grown at 37°C in lysogeny broth supplemented with 50-µg/mL neomycin at 150 rpm shaking for 3 h. Protein expression was then induced by addition of isopropyl β-d-1-thiogalactopyranoside to a final concentration of 0.2 mM, and the cells were incubated at 16°C overnight. Cells were harvested by centrifugation at 18,000 × *g* for 15 min, and then resuspended in 50-mM tris(hydroxymethyl)aminomethane (TRIS) buffer, pH 8, with 250-mM NaCl, 5-µg/mL lysozyme, and 10-µg/mL DNase I. Cells were lysed by sonication, and the soluble phase was collected by removing cell debris with centrifugation at 18,000 × *g* for 20 min. The resulting enzymes *As*CE15, *Pd*CE15, *Bi*CE15, and *Pv*CE15 were purified by immobilized metal ion affinity chromatography using 5-mL HisTrap Excel columns on an ÄKTA system (Cytiva). Binding buffer comprised 50-mM TRIS, pH 8, containing 250-mM NaCl, and the elution was performed using the binding buffer with the addition of 250-mM imidazole. *Pi*CE15A bound very tightly to any Sepharose-based resins which impeded any type of elution and was instead precipitated in 50% ammonium sulfate. The precipitate was resolubilized in binding buffer and loaded onto a HiLoad Superdex 200 16/60 gel filtration column and resolved with an isocratic gradient with binding buffer. All proteins were buffer exchanged into 50-mM TRIS, pH 8, buffer containing 100-mM NaCl by ultrafiltration using Amicon Ultra-15 centrifugal filtration units and stored at 4°C. Sodium dodecyl sulfate polyacrylamide gel electrophoresis using Mini-PROTEAN TGX Stain-Free Gels (Bio-Rad, Solna, Sweden) was used to verify protein purity, and protein concentrations were determined using a Nanodrop 2000 Spectrophotometer (Thermo Fisher Scientific, Waltham, MA, USA).

### Esterase activity determination using model substrates

**Fig 1:**
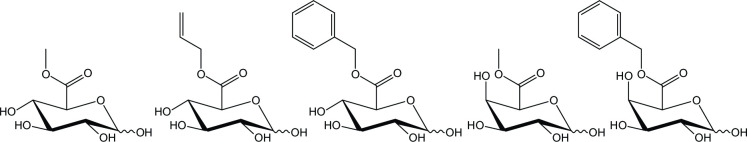
Structures of the model substrates used in the study. From left to right: methyl glucuronate, allyl glucuronate, benzyl-glucuronate, methyl galacturonate, and benzyl galacturonate.

The uronoyl esterase activity was assayed by continuously monitoring the formation of uronic acid using the K-URONIC kit (Megazyme). The pH optimum for each enzyme was determined with 1-mM benzyl-d-glucuronate (BnzGlcA) as a substrate in a constant ionic strength three-component buffer containing 50-mM TRIS-HCl, 25-mM acetic acid, and 25-mM 2-(*N*-morpholino)ethanesulfonic acid (MES), covering a pH range of 4.5–9.5 (45). Benzyl galacturonate (BnzGalA) was custom synthesized by Biosynth, UK (formerly Carbosynth). The kinetic measurements using BnzGlcA, allyl glucuronate (AllylGlcA), methyl glucuronate (MeGlcA), methyl galacturonate (MeGalA), and BnzGalA were performed as described by Arnling Bååth et al. (16) with the exception of the temperature, here set at 37°C. The substrates were dissolved in 100% dimethyl sulfoxide (DMSO), and all reactions contained ≤10% DMSO, an amount determined to not affect the enzyme reactions. Structures of the model substrates are shown in Fig. 1.

### Pectate lyase coupled assays

Pectin methyl esterase (PME) activity was detected by coupled assays using the pectate lyase family 1 enzyme from *Aspergillus niger* (*An*PL1, Megazyme: E-PCLYAN), an enzyme only capable of cleaving unmethylated galacturonan. Assays of 200 µL contained 0.035% pectin (various types supplied from Megazyme, K-PECID) or 1% poly-methylgalacturonan (Biosynth) in 100-mM potassium phosphate buffer at pH 7, 225 U of *An*PL1 (Megazyme), and were supplemented with or without 0.5 nmol of *Pv*CE15 or 1:100 of the PME NovoShape from *Aspergillus aculeatus* (University of Reading, UK). Pectate lyase product formation was observed by measuring the absorbance at 235 nm using a UV-Star UV transparent 96-well microplate (Greiner Bio-One) as described previously (46).

### Pectin-rich biomass saccharification assays

Biomass samples (carrot pomace, potato peel, and orange peel) were prepared by first homogenizing in a kitchen blender before lyophilization and subsequent ball milling. Ball-milled material (500 mg) was washed by resuspending in water (15 mL), mixing for 2 min, and with the solid material subsequently separated by centrifugation (5,000 × *g*, 10 min). After four washes, the material was resuspended in water to 50 mg/mL to be used immediately in assays. Assays of 2 mL, performed with four replicates, contained 5 mg/mL of biomass in 20-mM sodium phosphate, pH 7.5, and were treated with 1:2,000 of the enzyme cocktail Ultraflo L (Novozymes) supplemented with or without, or in combination, with 0.05 nmol of *Pv*CE15, 22.5 U of *An*PL1 (Megazyme), and 1:2,000 of the PME NovoShape (University of Reading). Reactions proceeded at 30°C with mixing at 750 rpm (Eppendorf ThermoMixer C) for 1 h and were stopped by heating at 95°C for 2 min, followed by cooling on ice for 2 min. Solid material was separated by centrifugation (15,000 × *g*, 2 min). Released monosaccharides in the aqueous fraction were quantified by high-performance anion exchange chromatography with pulsed amperometric detection on an ICS-5000 system equipped with a 4 × 250 mm Dionex Carbopac PA1 column with a 4 × 50 mm guard column maintained at 30°C, (Dionex, Sunnyvale, CA, USA). Ten-microliter samples were injected, and the eluents were A, water; B, 300-mM sodium hydroxide; and C, 100-mM sodium hydroxide and 85-mM sodium acetate. The samples were eluted isocratically with 100% A for 25 min (1 mL/min) and detected with post-column addition of 0.5 mL/min of 100% B. Thereafter, a cleaning step with 60% B and 40% C was performed at 1 mL/min for 10 min. Peak analysis was performed using Chromeleon software, and peaks were quantified against monosaccharide standards.

### Crystallization and structure determination of *Pv*CE15

Both *Pv*CE15 and *Pi*CE15A were screened for crystallization in MRC 2-drop crystallization plates (Molecular Dimensions) using an Oryx 8 Robot (Douglas Instrument), though only *Pv*CE15 proved amenable to crystallization. Sitting drops (0.3 µL) were mixed with protein:reservoir volume ratios of 3:1 or 1:1 using 20 mg/mL of protein prepared in 50-mM TRIS, pH 8, with 100-mM NaCl. Hits from a PACT Premier screen (Molecular Dimensions) were optimized, and final crystallization conditions yielding plate-shaped crystals contained 0.1-M MMT buffer at pH 9.0 (composed of DL-malic acid:MES:Tris base in a 1:2:2 ratio) and 25% (wt/vol) PEG 1500. For ligand-soaked structures, crystals were produced in a modified condition comprised of 0.1-M MES, pH 6.0, 20-mM NaCl, and 20%–24% (wt/vol) PEG 6000. For the GlcA complex structure, BnzGlcA powder was added directly to the drop containing the crystals and left to soak for 4 min. For the GalA soak, 0.5 µL of 0.25 M of neutralized GalA was added directly to the drop and left to soak for 3 min. Crystals were flash frozen in liquid nitrogen without the addition of cryoprotection. Data sets for the apo and GalA soak were collected on the BioMAX beamline (47) at MAXIV Lab, Lund, Sweden, and the data set for BnzGlcA soak was collected on ID30B (48) at the European Synchrotron Radiation Facility, Grenoble, France. Data collection at the synchrotrons was carried out using the MxCUBEv3 (49) software and the auto-processed results from the pipelines were tracked and analyzed using ISPyB (50). Diffraction data of the apo data set was processed with XDS (51), while the auto-processed data sets from the pipeline autoPROC_staraniso were used for structure determination of the GlcA and GalA data sets. Structure solutions were completed in Phenix (52), and the *Pv*CE15 structure was determined by molecular replacement with PHASER (53) using the glucuronoyl esterase from *Solibacter usitatus* (*Su*CE15C, Protein Data Bank identifier, PDB: 6GRY) as a search template. A model was initially built with Phenix AutoBuild (54), manually rebuilt in COOT (55), and further refined with Phenix Refine (56) in alternating cycles. The ligand-soaked data sets were phased by isomorphous difference Fourier methods using the native *Pv*CE15 structure. The ligand-soaked structures were refined by rigid body and non-crystallographic restrained refinement in REFMAC5 (57) of CCP4 suite (58) alternated with manual model building in COOT. For the BnzGlcA soak, the aromatic moiety was not visible in the electron density; thus, the GlcA product was modeled. The crystallographic statistics of the data sets are provided in Table S2.

## RESULTS

### PUL analysis and sequence comparisons

**Fig 2:**
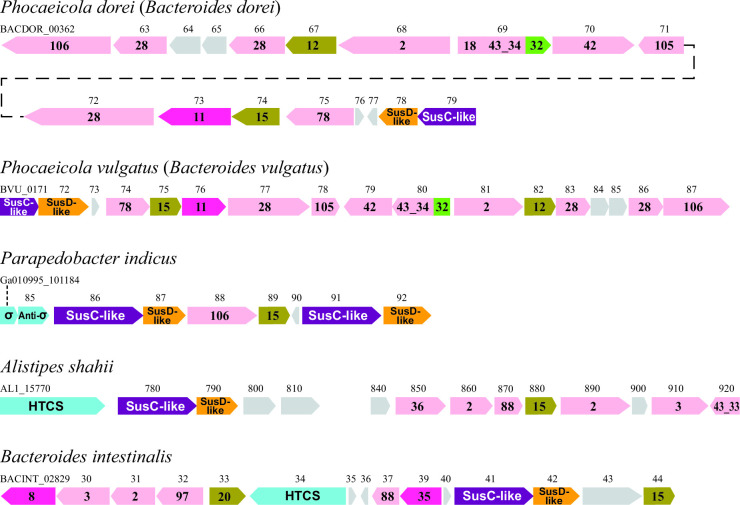
Overview of the organization of the PULs encoding the investigated CE15 enzymes, as predicted by the PULDB (35). Encoding species are listed above each PUL (with former names in parentheses where applicable), and locus tags are above each corresponding gene, drawn to scale. The top two PULs appear to target pectin; the middle might possibly target pectin-derived oligosaccharides, and the bottom two are unlikely to target pectin. Functional annotations are shown inside each gene symbol and are color coded following the PULDB color scheme: glycoside hydrolases in pink, proteins of unknown function in light gray, SusD-like binding proteins in orange, SusC-like transport proteins in violet, regulatory hybrid two-component systems and other regulatory factors (HTCSs, σ) in light blue, carbohydrate esterases in brown, polysaccharide lyases in purple, and carbohydrate-binding modules in green.

When investigating the PULDB (35), with CE15 as search term, several PULs not appearing to target xylan were identified, e.g., not encoding canonical GH10/11 endo-xylanases or GH43/51 β-xylosidases/α-l-arabinofuranosidases. The five chosen species—*A*. *shahii*, *B. intestinalis*, *P. indicus*, *P. dorei*, and *P. vulgatus—*all encode a singular CE15 gene copy apart from *P. indicus* which encodes five; the enzymes were named *As*CE15, *Bi*CE15, *Pd*CE15, *Pv*CE15, and *Pi*CE15A, respectively (Fig. 2). The CE15 containing PULs from *P. indicus*, *P. dorei*, and *P. vulgatus* appear to target pectin or pectin-derived oligosaccharides on the basis of encoding a variable number of putative enzymes with signature predicted functionalities, such as glycoside hydrolase family (GH) 28 (polygalacturonase/rhamnogalacturonase), GH78 and GH106 (α-l-rhamnosidase), GH105 (unsaturated rhamnogalacturonyl hydrolase), polysaccharide lyase family 11 (rhamnogalacturonan lyase), and carbohydrate-binding module family 32 (polygalacturonan-binding) (11). In part, they overlap in activities with previously identified PULs from *Bacteroides thetaiotaomicron* targeting RG-I (59). Curiously, the *P. indicus* PUL only encodes one GH106 enzyme in addition to the CE15 target, and it therefore likely does not have the functional breadth to be responsible for conversion of complex pectin. In contrast, the PULs from *A. shahii* and *B. intestinalis* appear to target some other heteropolysaccharide, such as chondroitin or gellan, based on the presence of genes encoding GH88 enzymes (unsaturated chondroitin/gellan glucuronosyl hydrolase). However, they also encode polyspecific GH family members, such as GH2 and GH3 (various β-glycosidases) and PL8 (hyaluronate/chondroitin/xanthan/heparin lyase), making the prediction of the target of the PULs uncertain.

In LCCs, esters are formed between GlcA moieties on xylan and lignin alcohol moieties, so the reason for the presence of CE15 enzymes in the aforementioned PULs is not obvious. Possibly, the CE15 family comprises not only GE activity but also other activities found in known families, or these enzymes target some as-of-yet unidentified linkage either in plant cell walls or other mammalian or even microbial polysaccharides. Primary sequence analysis reveals identity ranging from 21% to 52% of the here studied enzymes with those of some previously characterized fungal and bacterial enzymes (Table S3). When comparing with each other, the enzymes of this study are diverse in sequence, with *Pi*CE15A being the least similar to the others, having its highest sequence identity to *As*CE15 (42%). *Pd*CE15 and *Pv*CE15 appear almost identical, with a sequence identity of 99%, while *Bi*CE15 and *As*CE15 are also quite similar, sharing 59% sequence identity.

### Biochemical characterization

All protein targets were recombinantly produced in *E. coli* and purified to homogeneity (Fig. S1). The produced proteins were assayed with BnzGlcA, a model substrate for GE activity, and were all active. The pH dependence was determined using BnzGlcA and revealed similar optima among the enzymes, which preferred slightly alkaline conditions ranging from pH 7.5 to 8.5 (Fig. S2), characteristic of most previously characterized bacterial GEs (16). Full kinetic parameters of the five enzymes were then determined, where possible, using a variety of model substrates at pH 7.5, a pH value at or close to the optimal where at least 75% of each enzyme’s maximum activity was maintained (Table 1).

**TABLE 1:** Summary of kinetics of the studied CE15 enzymes with model substrates

Enzyme	Substrate	*K*_m_ (mM)	*k*_cat_ (s^−1^)	*k*_cat_/*K_m_* (/s^−1^/mM^−1^)
*As*CE15	BnzGlcA	2.63 ± 0.45	31.57 ± 1.63	11.99 ± 2.12
	MeGlcA	2.91 ± 0.48	19.85 ± 0.87	6.82 ± 1.17
	AllylGlcA	3.06 ± 0.43	21.17 ± 0.79	6.91 ± 1.00
	MeGalA	9.03 ± 2.08	1.35 ± 0.12	0.15 ± 0.04
	BnzGalA	6.26 ± 0.42	1.92 ± 0.04	0.31 ± 0.02
*Pd*CE15	BnzGlcA	0.91 ± 0.12	120.07 ± 3.98	131.79 ± 17.8
	MeGlcA	1.27 ± 0.12	122.32 ± 3.25	96.59 ± 9.61
	AllylGlcA	1.05 ± 0.11	133.54 ± 3.65	127.47 ± 13.77
	MeGalA	4.83 ± 0.25	39.33 ± 0.76	8.14 ± 0.45
	BnzGalA	1.18 ± 0.09	72.38 ± 1.75	61.49 ± 4.86
*Bi*CE15	BnzGlcA	1.68 ± 0.27	77.04 ± 3.38	45.88 ± 7.59
	MeGlcA	2.26 ± 0.20	57.65 ± 1.69	25.55 ± 2.37
	AllylGlcA	2.37 ± 0.29	63.92 ± 2.76	26.95 ± 3.5
	MeGalA	Could not be saturated		0.14 ± 0.011
	BnzGalA	Could not be saturated		0.35 ± 0.028
*Pv*CE15	BnzGlcA	0.73 ± 0.09	96.66 ± 3.42	131.63 ± 16.33
	MeGlcA	1.44 ± 0.16	78.83 ± 2.43	54.62 ± 6.10
	AllylGlcA	0.97 ± 0.06	86.38 ± 1.75	89.18 ± 5.91
	MeGalA	5.70 ± 0.29	36.33 ± 0.93	6.37 ± 0.37
	BnzGalA	1.07 ± 0.09	49.01 ± 1.20	45.66 ± 4.01
*Pi*CE15A	BnzGlcA	0.57 ± 0.11	6.73 ± 0.29	11.86 ± 2.38
	MeGlcA	3.83 ± 0.64	4.99 ± 0.35	1.3 ± 0.23
	AllylGlcA	1.71 ± 0.17	6.48 ± 0.19	3.8 ± 0.38
	MeGalA	2.49 ± 0.49	2.59 ± 0.14	1.04 ± 0.21
	BnzGalA	0.54 ± 0.05	5.11 ± 0.10	9.49 ± 0.87

All five enzymes exhibited a catalytic efficiency on BnzGlcA similar to that of previously characterized bacterial GEs (16, 60, 61), with *Bi*CE15 and *Pv*CE15 showing the highest efficiency with *k*_cat_/*K_m_* values in the 10^5^/s^−1^/M^−1^ range. The *K_m_* values for BnzGlcA were also comparable with the ones observed in many previously characterized bacterial GEs, being in the low millimolar range. Noteworthy are the *K_m_* values measured for *Pv*CE15 and *Pi*CE15A both reaching a submillimolar range of 0.73 and 0.57 mM, respectively. None of the enzymes from *A. shahii*, *P. dorei*, *B. intestinalis*, or *P. vulgatus* showed major decreases in efficiency when cleaving GlcA-based substrates with different substituents, from benzyl to allyl (AllylGlcA) and methyl (MeGlcA), with *As*CE15, *Bi*CE15, and *Pv*CE15 all showing a maximum of twofold decrease in *k*_cat_/*K_m_*, and the catalytic efficiency of *Pd*CE15 appeared to be more or less unvaried across the three different substrates, suggesting deacylation as the rate-limiting step. It is worth to point out that the observed differences for *Pd*CE15, *Bi*CE15, and *Pv*CE15 were mostly attributable to an increase in *K_m_* values rather than decreases in *k*_cat_ values, though the opposite was the case for *As*CE15. These results would suggest that the nature of the ester substituents does not have a major effect on the overall activity of these enzymes, but all have a general preference for bulkier substituents. *Pi*CE15A, on the other hand, showed a decrease of the catalytic efficiency of threefold on AllylGlcA and of an entire order of magnitude on MeGlcA relative to the preferred BnzGlcA substrate, indicating the nature of the alcohol substituent plays a significant role for the activity of the enzyme and thus that acylation is the rate-limiting step. When switching from glucuronate to galacturonate methyl esters (MeGalA), all enzymes exhibited a decrease in catalytic efficiency of up to an order of magnitude, with the exclusion of *Pi*CE15A, which interestingly did not discriminate between the two uronic acid substrates.

The hypothesis that LCCs could potentially be formed between lignin and pectin prompted us to obtain the GalA analog of BnzGlcA, a compound not previously commercially available. Similar to the activity with MeGalA, both *As*CE15 and *Bi*CE15 had poor activity with BnzGalA, corresponding to a decrease of two orders of magnitude compared to their respective efficiency on BnzGlcA, which could be related to their localization in PULs with no apparent connection to pectin or GalA-rich polysaccharides. *Pd*CE15 and *Pv*CE15 also exhibited a decreased efficiency on BnzGalA compared to that with BnzGlcA, but only a difference around twofold was observed and mainly resulting from reduced *k*_cat_ values. Once again, *Pi*CE15A stood out as an interesting family member in that it showed no apparent discrimination between the two substrates, suggesting that for this enzyme, the nature of the alcohol moiety is more important than that of the uronic acid residue for substrate recognition and hydrolysis.

### Investigation of enzymes with pectin and pectin-rich biomasses

**Fig 3:**
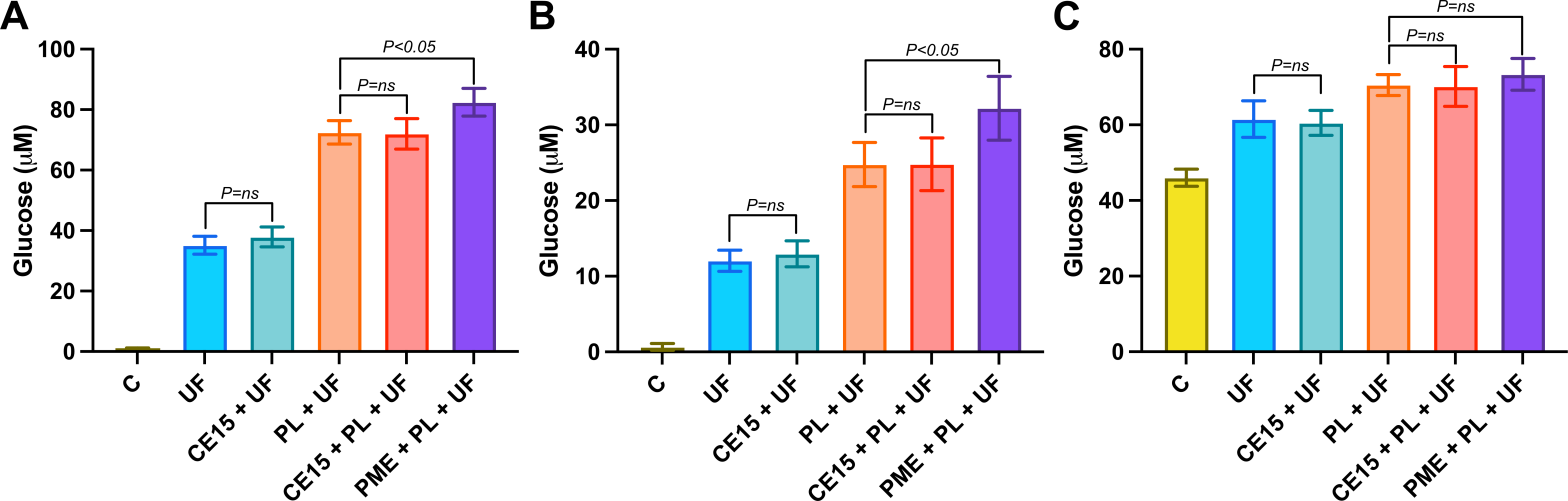
Effect of *Pv*CE15 on glucose release during a 1-h saccharification of certain pectin-rich biomasses. Biomass materials of (**A**) carrot pomace, (**B**) potato peels, and (**C**) orange peels were milled, washed, and resuspended in assays of 5 mg/mL and incubated for 1 h at 30°C with either UltraFlo (UF), *Pv*CE15 (CE15), pectate lyase (PL), or pectin methyl esterase (PME) and were compared to control reactions (**C**) lacking the addition of enzymes. Released monosaccharides from assays (*N* = 4) were quantified by ion chromatography, and the differences in means were evaluated by Student’s *t*-test. The addition of *Pv*CE15 to either UltraFlo or UltraFlo supplemented with the pectate lyase did not lead to significant increases (ns) in released glucose, while the addition of PME to the reactions with UltraFlo supplemented with the pectate lyase did for both carrot pomace and potato peels but not orange peels. The washed orange peel control samples contained a considerable amount of free glucose that was not present before the 1-h incubation, indicating additional factors were contributing to monosaccharide release during the assays.

In contrast to what has been reported for fungal enzymes (13, 15), many previously studied bacterial GEs are able to hydrolyze MeGalA, though typically with decreased affinity and catalytic efficiency when compared to most other model substrates (10, 16). However, the position of these enzymes in putative pectin-targeting PULs merited the investigation of whether they could remove methyl-ester substituents from GalA moieties in homogalacturonan (poly-methylgalacturonan), thus acting as PMEs, as hypothesized during the bioinformatic analysis. Esterified rhamnogalacturonan is, however, not commercially available to our knowledge, precluding comparisons on a heteropolysaccharide. Assays coupling PME activity to pectate lyase activity were employed, with pectin substrates differing in biological sources and varying in their degree of methylation using *Pv*CE15, one of the more efficient enzymes. However, the addition of *Pv*CE15 to pectate lyase *An*PL1 reactions did not increase pectin cleavage, while the addition of a bona fide PME from *Aspergillus aculeatus* did. We additionally explored if these enzymes could enhance the saccharification of pectin-rich plant biomass, similar to how GEs previously have been shown to boost hydrolysis of xylan-rich biomass (9, 16). The enzyme saccharification cocktail UltraFlo was used in 1-h assays to hydrolyze pre-washed carrot pomace, potato peels, and orange peels, which may have ~3% to 30% pectinaceous dry matter content (17). The released monosaccharides were subsequently quantified. The addition of a pectate lyase clearly improved the release of glucose from these biomasses, and inclusion of the PME further improved the release (Fig. 3). However, addition of *Pv*CE15, either directly to the Ultraflo cocktail or in further combination with the pectate lyase, did not show any significant increase in glucose release. Similar differences in the amount of arabinose released was also observed, but the variance in those results makes it difficult to draw definitive conclusions (Fig. S3). Notably, released amounts of xylose, galactose, and mannose were consistently below quantification limits (2.5 µM) and are not shown here. The amount of monosaccharide uronate (glucuronate or galacturonate) released by the reactions was quantified by coupling free uronate to NAD^+^ reduction by uronate dehydrogenase, but notably, none of the reactions produced uronate above the 5-µM detection limit.

### Protein structure of *Pv*CE15

**Fig 4:**
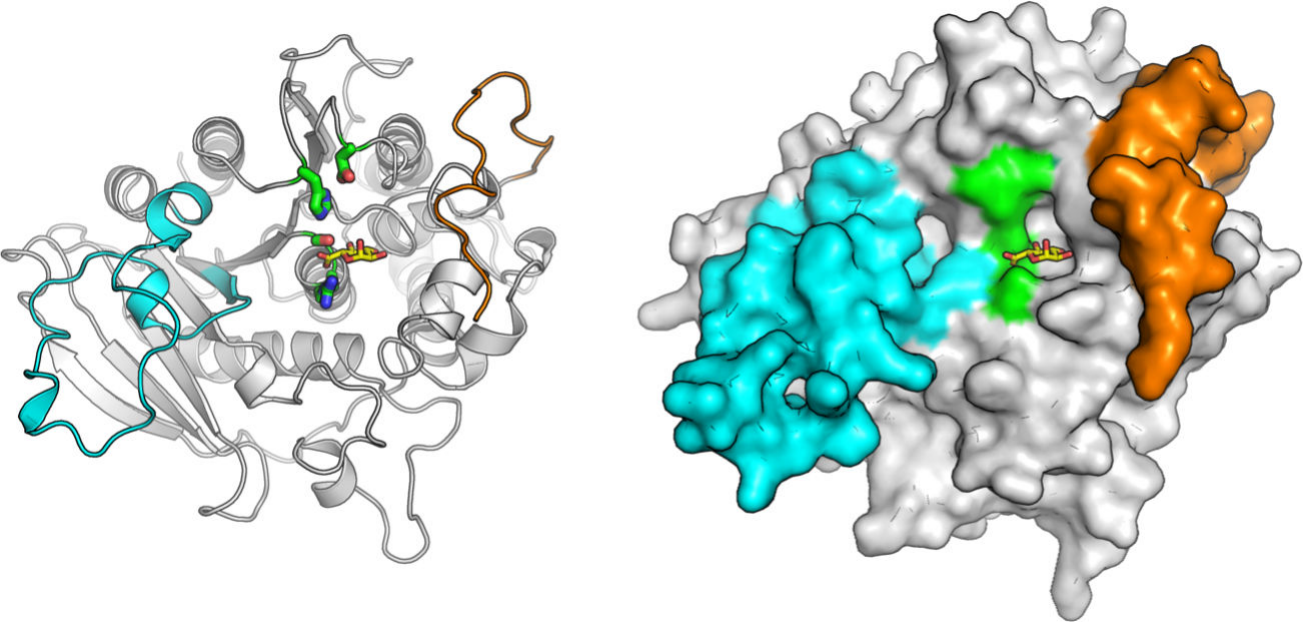
Overall structure of *Pv*CE15. A cartoon representation of *Pv*CE15 (left, PDB accession: 8Q6S) in complex with GlcA, shown in yellow sticks, with the residues of the catalytic triad shown as green sticks and the regions corresponding to Reg2 and RegN shown in cyan and orange, respectively. A surface representation (right) illustrating the buildup of the binding cleft by Reg2 and RegN.

**Fig 5:**
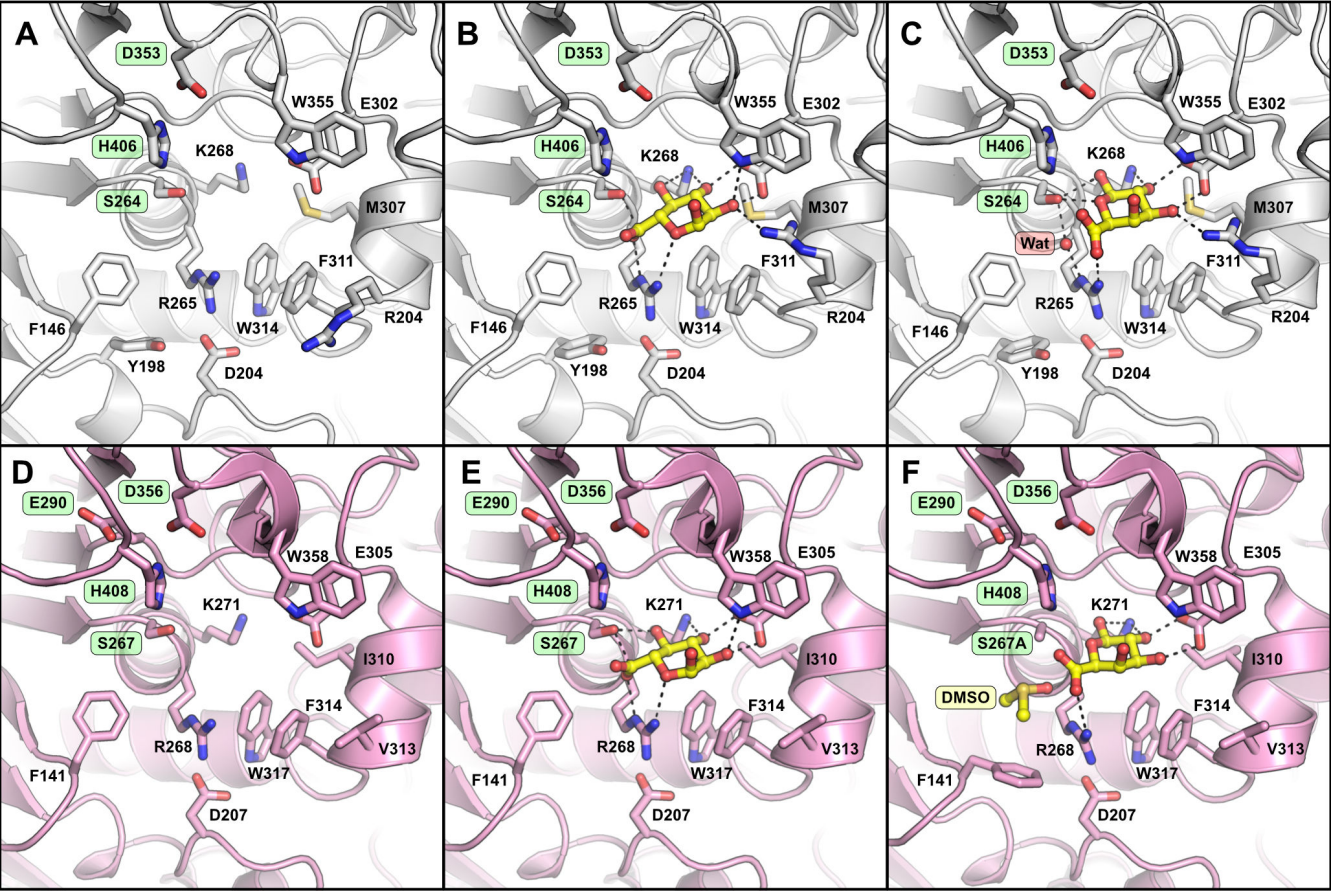
Active site organization of *Pv*CE15 in complex with uronate substrates. The active site of *Pv*CE15 (A, PDB accession: 8Q6S) in complex with glucuronate (B, PDB accession: 8QCL), and galacturonate (C, PDB accession: 8QEF) compared to the active site of *Ot*CE15A (D, PDB accession: 6GS0) in complex with glucuronate (E, PDB accession: 6SYR) and galacturonate (F, PDB accession: 6SZO). Residues of the catalytic triad and the additional catalytic acidic residue in *Ot*CE15A are highlighted in green, and the water and DMSO molecules found in the galacturonate complex structures are highlighted in red and yellow, respectively. The galacturonate complex with *Ot*CE15A was obtained with the catalytic serine substitution (S267A). Key interaction distances ≤3 Å are shown as black dashes.

To gain greater insights into the structural determinants defining uronate specificity in CE15, we pursued protein structures of both *Pv*CE15 and *Pi*CE15A, as they had similar specificities for GlcA and GalA esters. Only *Pv*CE15 proved amenable to crystallization, and its structure was solved both as an apo protein and in complex with GlcA and GalA, respectively. Crystals of the apo and ligand-soaked structures were both of space group P2_1_ and contained two molecules in the asymmetric unit with similar cell dimensions. *Pv*CE15 has an α-/β-hydrolase fold like previously determined CE15 structures, consisting of a three-layer sandwich with a solvent-exposed cleft comprising the active site (Fig. 4). A key feature distinguishing the bacterial and fungal CE15 members which have been structurally determined thus far is the presence of inserted regions present in the bacterial members (referred to as Reg2 and RegN (16, 61), which build up the active site cleft compared to the more solvent exposed active sites of fungal members, and such inserted regions are found also in *Pv*CE15 (Fig. 4). The N-terminal region (RegN) forms a loop which builds up one side of the binding cleft in a similar position to the RegN observed in *Tt*CE15A (61), though the latter is longer and extends beyond the binding cleft. The region corresponding to Reg2 in *Pv*CE15 is a long loop partially protruding from the structure with its residues having higher temperature factors compared to the core structure. There is considerable diversity in length and sequence identity in Reg2 among bacterial members, and they have been proposed to be involved in either recognition or tolerance of larger lignin polymers or fragments (16, 61, 62). Most insert regions are predominantly hydrophilic, and most contain a phenylalanine (Phe146 in *Pv*CE15) proximal to the catalytic residues that possibly provide specificity toward the alcohol portions of the ester substrates, such as lignin fragments (Fig. 5). The catalytic triad (Asp353-His406-Ser264) and oxyanion-stabilizing arginine (Arg265, oxyanion hole), whose positions and roles have been established in previous work on CE15 GEs (60, 61, 63, 64), are conserved in *Pv*CE15 (Fig. 5). Conserved are also the highly conserved residues supporting glucuronate binding within CE15, such as the tryptophan, glutamate, and lysine residues found interacting with the glucuronate O2, O2 and O3, and O4 hydroxyls, respectively (Trp355, Glu302, and Lys268).

Attempts to obtain a Michaelis complex with the substrate BnzGlcA were unsuccessful but afforded GlcA complexes in both protomers in the asymmetric unit with the ligand bound in the same orientation as other GE-GlcA complexes (Fig. 5). A notable distinction from previously determined CE15 members is the presence of an arginine residue in *Pv*CE15 found at the edge of the binding cleft whose side chain, found projecting away from the pocket in the native structure, is rotated inward with its guanidinium positioning the glucuronate O2. A complex structure with GalA was obtained by soaking with neutralized GalA, which revealed a binding pose distinct from the GlcA-binding pose, where the pyranose ring is flipped, resulting in the anomeric hydroxyl pointing into the cleft and positioned where the GlcA O4 is found (Fig. 5). Distinctly, the carboxylate of the uronate is positioned out of the presumed catalytic center, with the C6 far from the catalytic serine (3.9 Å), but it is still positioned by the oxyanion-stabilizing arginine (Arg265) through one of its Nη atoms. A water molecule is found occupying the presumed catalytic center and is 3.2 Å away from the C6 of the uronate, which could suggest a water-mediated mechanism for this substrate. This orientation for GalA has also been observed with *Ot*CE15A from *Opitutus terrae* (60, 64), which has a similar preference for MeGlcA and MeGalA substrates, possibly suggesting a water-mediated mechanism for GalA substrates that is distinct from the mechanism utilized for GlcA substrates. However, the complexes obtained are only of the products of the reactions which may bind in a different orientation than required for catalysis.

The electrostatic charge or hydrophobic characteristics of a protein’s surface can offer insights into the type of molecules that an enzyme might interact with. For instance, an electropositive surface near a binding site could be conducive to binding with electronegative substrates such as galacturonan, as demonstrated in certain polygalacturonases (65, 66). When examining the surface charge and hydrophobicity of *Pv*CE15, a small electropositive patch is seen across the ridge leading away from the sugar-binding site and catalytic residues (Fig. S4A). However, this patch is not very prominent, principally resulting from a single lysine residue, and the patch is not conserved among predicted models of other enzymes from suspected pectin PULs investigated in this study (Fig. S4B). Note that while the presence of specific electropositive areas could suggest potential sites for binding with electronegative substrates, the absence of such regions around the active site does not necessarily rule out the possibility of the enzyme targeting such substrates.

## DISCUSSION

The CE15 enzymes investigated in this study were found in predicted PULs containing putative enzymes belonging to families not expected to target xylan, which is surprising, given previous knowledge on GEs and their roles in cleaving xylan-lignin LCC bonds. Instead, the genetic organization suggests these PULs target either pectic polysaccharides or other glycans, leading to the hypothesis that their encoded CE15 enzymes would act on uronate-based esters on non-xylan polysaccharides. During the course of this work, the demonstration of pectin-lignin LCCs was published (25), which may shed light on the roles of some of the enzymes studied here. Complex carbohydrates are likely major nutrient sources for the five chosen species from which we sourced the enzymes, coming from gut environments except the soil bacterium *P. indicus* (41). *B. intestinalis*, *P. dorei*, and *P. vulgatus* have been shown to grow on xylan previously (42–44), but for the other strains, information is lacking; analysis of the genome of *A. shahii* reveals no obvious xylanases, and similarly in *P. indicus*, there is a lack of a predicted abundant xylanolytic machinery with only two putative GH10 xylanases apparently present. It is thus not impossible that some of the enzymes investigated here would target LCC bonds between glucuronoxylan and lignin *in vivo*, but it is unlikely due to their placement in PULs, which target other complex polysaccharides. Through a combination of biochemical and structural work, we have tried to piece together some characteristics of a plausible natural substrate.

First, the results produced here conclusively show that backbone esterified uronic acids are not targeted by these CE15 enzymes. From the onset, analysis of previously determined CE15 structures indicated that accommodating a main chain uronic acid would be extremely challenging, given the extensive interactions of the sugar acid within the binding pocket. We considered the possibility that these enzymes might bind GlcA and GalA in distinct ways which could then facilitate action upon backbone residues. To investigate this, we obtained crystal structures of *Pv*CE15 in complexes with both ligands. Although observed to bind in different orientations, neither orientation could easily be envisioned to support uronic acid being part of a main chain with linkages at the O1 and O4 positions, as is the case in the galacturonan backbone. Among the PULs examined here which contain pectin targeting members, none encode an obvious PME candidate. While the structural analysis indicated a lack of activity on backbone residues, the significant activity demonstrated by certain GEs on MeGalA prompted us to investigate the initial hypothesis that these CE15 members could have PME activity to support the PULs. However, no PME activity was observed on isolated pectins. There was also no boosting of the pectate lyase cleavage of isolated pectins nor was there any boosting of saccharification of pectin-rich biomass when supplementing an enzyme cocktail, in contrast to that seen when using a bona fide PME. Taken together, the results indicate that these CE15 enzymes neither have canonical PME functionality nor do they act on main chain uronic acids, but rather must act on terminal or branching moieties.

Generally, CE15 enzymes seem to divide in two groups: one discriminating strongly between GlcA and GalA esters, including enzymes with virtually zero activity on GalA esters, and the other, which has comparable activities on both model substrates. The biochemical characterization on the model substrates showed that the studied CE15 enzymes have similar kinetic parameters to that of previously characterized family members (16, 60, 61) and that all of the five enzymes investigated here were active on MeGalA, although all of them except for *Pi*CE15A have a decrease of at least 10-fold in the catalytic efficiency compared to MeGlcA. Such dual-specificity CE15 enzymes may be utilized in nature on complex substrates containing both GlcA/GalA esters. For example, methyl esters of side-chain GlcA moieties are reported in RG-II (67) and could potential exist in other pectic polysaccharides, such as terminal GlcA that have been reported in the arabinogalactan II portions of RG-I (68). To the best of our knowledge, methyl esters of the side-chain GalA units of RG-II have not been reported but their methylation is conceivable. Thus, these dual GlcA/GalA utilizing CE15 members within the pectin targeting PULs could be utilized to target these terminal or sidechain uronate methyl esters. This notion is supported by the presence of GH106 members in all three of the putative pectin-targeting PULs described here, genes normally associated with l-rhamnosidase activity, which could act on l-rhamnose units linked to the sidechain GalA units (67). Furthermore, the previously reported *Ot*CE15C (16) consists of a fused CE15 and GH106 domain, where the CE15 domain interestingly also has a dual MeGlcA/MeGalA specificity, but the GH106 has thus far not been characterized. There are also some CE15 members which, although containing a seemingly competent active site and binding cleft (69), lack activity on model substrates (69, 70), which could suggest that members of the CE15 family contain a discrete specificity for linkages unobserved previously.

Covalent pectin-lignin connections have been proposed as well as recently shown in literature (21, 24, 25), where uronic acid-mediated linkages can be envisioned, similar to linkages between glucuronoxylan and lignin, and these remain a possible target of the enzymes studied here. Like previously characterized bacterial GEs (16, 40, 61, 71), the enzymes investigated in this study have a slight preference for BnzGlcA over MeGlcA. To probe bonds to GalA, we used the new substrate BnzGalA, which contains a bulky alcohol moiety like BnzGlcA. All the enzymes showed a preference for the bulkier BnzGalA compared to MeGalA, with the BnzGalA activity being comparable to the activity for BnzGlcA, especially for *Pi*CE15A, where the activities were within error of the assays. This provides some support for the idea that some of the characterized enzymes could detach lignin connected to branching or terminal uronic acids, for instance, on the non-reducing end of HG. Interestingly, the enzymes with the lowest activity on GalA-based substrates were those found in PULs with no clear connection to pectin metabolism, i.e., *As*CE15 and *Bi*CE15. Evaluating the action of our discovered new enzymes, or close homologs thereof, on material with proven pectin-lignin bonds (25) will be a highly promising route forward to conclusively verify their biological roles. An alternative approach to unravel the biological roles of these enzymes would be to investigate which specific poly- or oligosaccharides trigger a PUL’s upregulation by cultivating each species on pure carbohydrates and coupling this to transcriptomic analyses (29). As PULs are upregulated upon the bacterium sensing specific carbohydrate motifs, i.e., certain degradation products, whether the PULs used here as enzyme sources are triggered by the same or different glycans could be determined.

The complex structure of *Pv*CE15 with GalA is found in a binding pose distinct from that of GlcA, where the carboxylate is positioned out of the catalytic center and could suggest a water-mediated mechanism for this substrate distinct from the serine adduct previously observed with *Ot*CE15A and proposed for other CE15 members with GlcA substrates (60, 72). The analogous pose was also observed in *Ot*CE15A using a catalytically crippled variant, where the catalytic serine was replaced with alanine (S267A), and the difference in GalA versus GlcA positioning was speculated to be an artifact of residue substitution or the presence of DMSO solvent molecules present (60). However, this new observation of GalA also with the wild type *Pv*CE15 reinforces the proposal of a distinct water-mediated mechanism for this substrate, especially for CE15 members with similar activity on GalA and GlcA esters. What remains unclear is how uronate specificity is governed in members of the family that show a strict specificity for GlcA or GalA esters, such as *Bi*CE15 studied here or the previously studied *Ot*CE15D, *Sl*CE15B, *Sl*CE15C, and *Su*CE15B (16). The structurally determined *Tt*CE15A also has a strict preference for GlcA versus GalA esters, though no obvious differences in residue identities or orientations are observed relative to other CE15 members to account for such the discrepancy in specificity (61). Future studies should seek to determine protein structures of more members containing this strict specificity to illuminate the molecular determinants defining uronate specificity.

In summary, we present the characterization of five new CE15 enzymes with either predominant GE activity with GalA esters or dual preference for GlcA/GalA-based esters. We exclude that these enzymes can act on main chain uronic acid esters. For the enzymes belonging to putative pectin-targeting PULs, we speculate that reported or yet unknown GlcA esters or yet unknown sites of lignin linkage onto GalA side-chain uronic acids could be the target bonds.

## Data Availability

Atomic coordinates and structure factors have been deposited at the RCSB Protein Data Bank with accession codes 8Q6S (PvCE15 apo structure), 8QCL (PvCE15 in complex with glucuronic acid), and 8QEF (PvCE15 in complex with galacturonic acid).

## SUPPLEMENTAL MATERIAL

Supplementary information aem.01768-23-s0001.pdf Tables S1-S3, Figures S1-S4, supplementary references.

ASM does not own the copyrights to Supplemental Material that may be linked to, or accessed through, an article. The authors have granted ASM a non-exclusive, world-wide license to publish the Supplemental Material files. Please contact the corresponding author directly for reuse.
