# Supplementary material for: Polysaccharide utilization loci from Bacteroidota encode CE15 enzymes with possible roles in cleaving pectin-lignin bonds
Source: Appl Environ Microbiol. 2024 Jan 5;90(1):e01768-23. doi: 10.1128/aem.01768-23 (PMC10807430; doi:10.1128/aem.01768-23)
Supplement: Supplementary information — Tables S1-S3, Figures S1-S4, supplementary references. [file aem.01768-23-s0001.pdf]

Supplementary information for:

Polysaccharide utilization loci from Bacteroidota encode CE15 enzymes with possible role in cleaving pectin-lignin bonds

Andrea Seveso[1], Scott Mazurkewich[1], Sanchari Banerjee[2], Jens-Christian Navarro Poulsen[2], Leila Lo Leggio[2], Johan Larsbrink[1]

[1]Wallenberg Wood Science Center, Division of Industrial Biotechnology, Department of Life Sciences, Chalmers University of Technology, SE-412 96 Gothenburg, Sweden

[2]Department of Chemistry, University of Copenhagen, Universitetsparken 5, DK-2100 Copenhagen, Denmark

Table S1. Primers used in this study.

Gene	Organism	Primer	5'-3' sequence
*As*CE15	*Alistipes shahii*	Forward	CAGATGCCCGCGACAAA
		Reverse	TCGTTTCAGGTGTTTGTCCG
*Pd*CE15	*Phocaeicola dorei*	Forward	GGGAAGTCTCCTAGAAAAGACTATG
		Reverse	TTTGTTTTTAAAATATTTGTCAGCAAAACG
*Bi*CE15	*Bacteroides intestinalis*	Forward	AGTCTTCCTAAAGACACTATCTTCAG
		Reverse	CTTTCTGAACCATTTATCTGCAAACT
*Pv*CE15	*Phocaeicola vulgatus*	Forward	GGAAAATCTCCTAGAAAAGACTATGC
		Reverse	TTTGTTTTTAAAATATTTGTCAGCAAAGC
*Pi*CE15A	*Parapedobacter indicus*	Forward	GGCCAGCCAGATCCC
		Reverse	TAAAAATTTTTTCATGTATTCAATAAATACCTGC

Table S2. Summary of crystallographic statistics.

	*Pv*CE15	*Pv*CE15-GlcA	*Pv*CE15-GalA
Data Collection			
Date	06/12/2019	09/09/2022	07/12/2022
Source	BioMAX at MAXIV	ID30B at ESRF	BioMAX at MAXIV
Wavelength (Å)	0.976254	0.9116	0.9763
Space group	$P2_1$	$P2_1$	$P2_1$
Cell dimensions			
a, b, c (Å)	77.4, 58.7, 89.5	74.3, 59.5, 87.5	74.3, 59.2, 87.3
α, β, γ (°)	90.0, 103.9, 90.0	90.0, 101.6, 90.0	90.0, 101.4, 90.0
No. of measured reflections	276530 (20598)	191728 (9957)	193217 (8592)
No. of independent reflections	50852 (4674)	42976 (2149)	28249 (1413)
Resolution (Å)	43.45 - 1.99 (2.06 - 1.99)	48.90 - 1.84 (1.99 - 1.84)	85.40 - 2.16 (2.20 - 2.16)
Ellipsoidal resolution limit (Å)[1]	-	2.239 [0.715 a* - 0.699 c*]	2.063 [0.740 a* + 0.672 c*]
	-	2.271 [b*]	2.494 [b*]
	-	1.730 [0.541 a* + 0.841 c*]	2.577 [-0.583 a* + 0.813 c*]
R_{merge}[2]	0.105 (2.146)	0.099 (0.827)	0.259 (1.183)
Completeness spherical (%)	94.16 (85.19)	65.7 (14.7)	70.6 (15.9)
Completeness ellipsoidal (%)	-	93.0 (75.2)	98.3 (70.9)
$CC_{1/2}$ (%)	0.998 (0.413)	99.6 (57.5)	98.6 (47.6)
$\langle I/\sigma(I)\rangle$	8.66 (0.64)	8.4 (1.6)	6.7 (1.7)
Multiplicity	5.4 (4.4)	4.5 (4.6)	6.8 (6.1)
Refinement			
R_{work}/R_{free}	0.194/0.258	0.179/0.231	0.184/0.254
No. of atoms			
Protein	6401	6339	6289
Ligand/ions	21	44	30
Water	281	289	97
Average B-factors			
Protein	45.3	27.9	35.6
Ligand/ions	56.5	40.4	48.6
Water	45.2	28.9	25.8
RMSD from ideal geometry[3]			
Bond lengths (Å)	0.010	0.013	0.013
Bond Angles (°)	1.12	1.76	1.83
Ramachandran Statistics			
Favoured/Allowed/Outliers (%)	95.9/4.0/0.1	96.4/3.4/0.3	94.7/4.8/0.5
PDB accession	8Q6S	8QCL	8QEF

[1] Brackets represent the direction along the reciprocal lattice. [2] $R_{merge} = \sum_{hkl}\sum_i |I_i(hkl) - \langle I(hkl)\rangle| / \sum_{hkl}\sum_i I_i(hkl)$, wherein $I_i(hkl)$ is the intensity of the *i*th measurement of reflection *hkl*, and $\langle I(hkl)\rangle$ is the mean value of $I_i(hkl)$ for all the *i* measurements. [3] Root mean square deviations from ideal geometry values (1).

Table S3. Sequence identity matrix of certain characterized CE15 members. Additional members beyond this study include the fungal GEs from *Cerrena unicolor* (*Cu*GE) and *Sporotrichum thermophile* (*St*GE2) highlighted in green, and bacterial CE15 members from *Opitutus terrae* (*Ot*CE15A and *Ot*CE15C), *Solibacter usitatus* (*Su*CE15C), and *Teredinibacter turnerae* (*Tt*CE15A) highlighted in red. Sequences were trimmed to only the CE15 domain, ie. excluding possible additional binding- or catalytic domains, and signal peptides.

	*As*CE15	*Pd*CE15	*Bi*CE15	*Pv*CE15	*Pi*CE15A	*Cu*GE	*St*GE2	*Ot*CE15A	*Ot*CE15C	*Su*CE15C	*Tt*CE15A
*As*CE15	100	51.4	60.5	51.4	42.0	25.8	25.3	52.7	44.4	48.8	34.8
*Pd*CE15	51.4	100	51.5	98.8	38.9	25.8	25.0	45.0	40.6	48.9	30.3
*Bi*CE15	60.5	51.5	100	51.3	37.5	27.0	24.4	45.9	38.6	46.3	30.5
*Pv*CE15	51.4	98.8	51.3	100	39.4	25.5	25.3	45.2	41.1	49.1	30.0
*Pi*CE15A	42.0	38.9	37.5	39.4	100	24.7	25.7	36.8	54.0	40.4	32.7
*Cu*GE	25.8	25.8	27.0	25.5	24.7	100	48.1	26.1	26.0	23.8	22.9
*St*GE2	25.3	25.0	24.4	25.3	25.7	48.1	100	26.3	25.2	24.5	22.3
*Ot*CE15A	52.7	45.0	45.9	45.2	36.8	26.1	26.3	100	43.9	48.6	32.6
*Ot*CE15C	44.4	40.6	38.6	41.1	54.0	26.0	25.2	43.9	100	45.6	34.2
*Su*CE15C	48.8	48.9	46.3	49.1	40.4	23.8	24.5	48.6	45.6	100	34.7
*Tt*CE15A	34.8	30.3	30.5	30.0	32.7	22.9	22.3	32.6	34.2	34.7	100

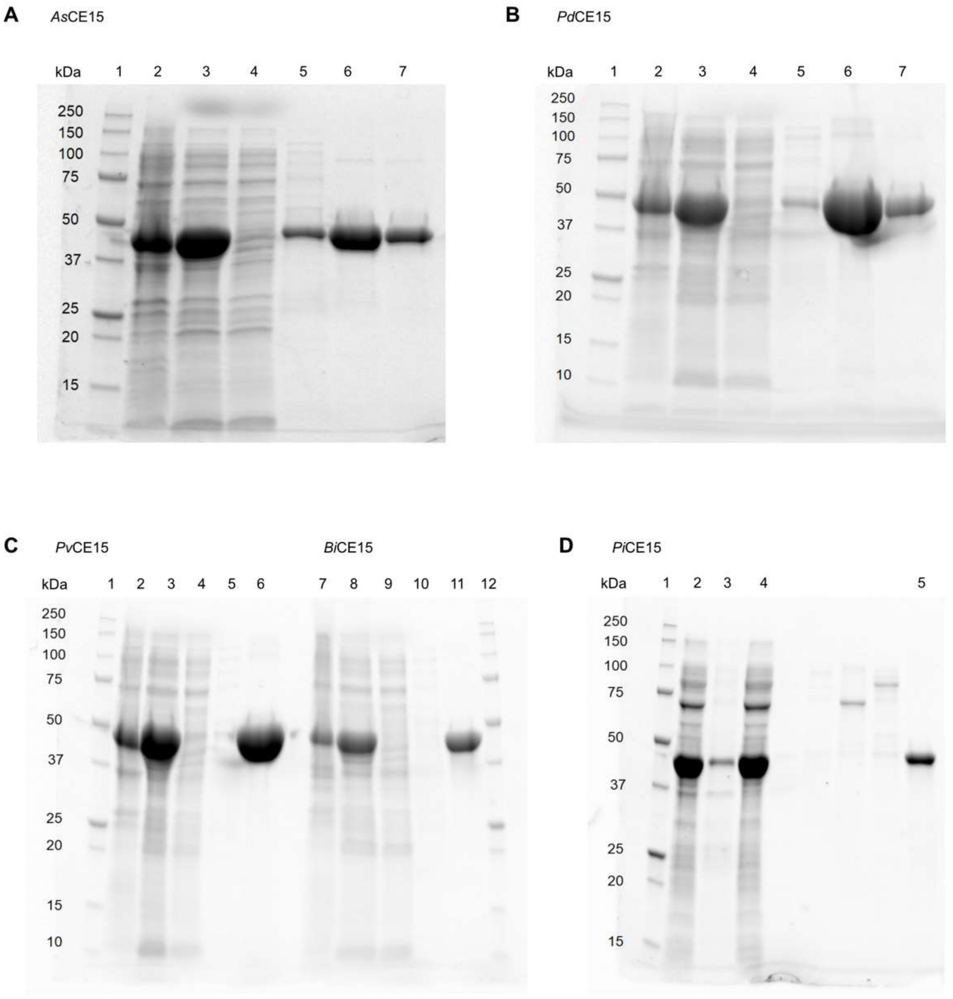

Figure S1. SDS-PAGE analysis of FPLC purification steps from *E. coli* cell lysates. A) Results from IMAC for *As*CE15 with marker (1), insoluble phase of cell lysate (2), soluble phase (3), flowthrough (4), wash (5), elution (6 and 7). B) Results from IMAC for *Pd*CE15 with marker (1), insoluble phase of cell lysate (2), soluble phase (3), flowthrough (4), wash (5), elution (6,7). C) Results from IMAC for *Pv*CE15 and *Bi*CE15 with marker (1, 12), insoluble phase of cell lysate (2,7), soluble phase (3,8), flowthrough (4,9), wash (5,10), elution (6,11). D) Results from SEC for *Pi*CE15A with marker (1), resuspension after ammonium sulfate precipitation (2), precipitate after centrifugation (3), soluble phase (4), elution (5), and with unlabeled lanes corresponding to other peaks in the chromatogram that were analyzed to verify the absence of *Pi*CE15A.

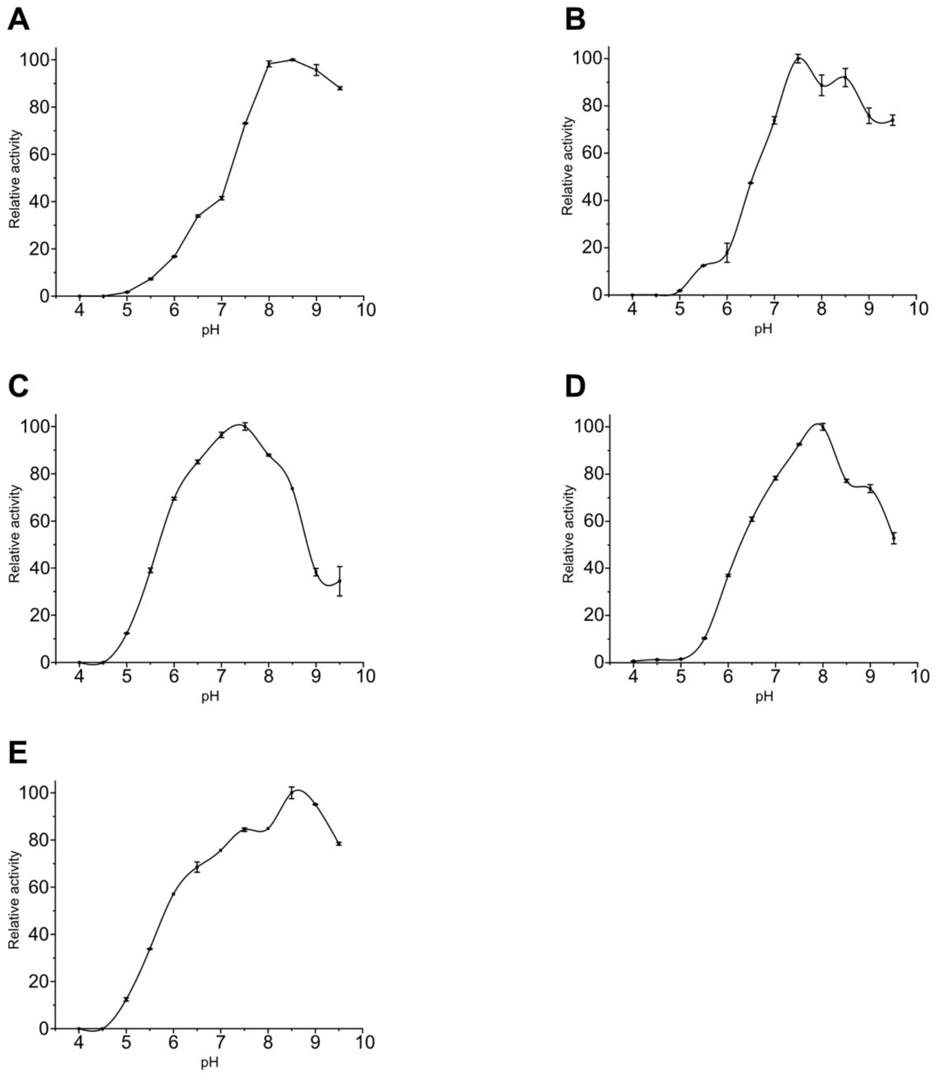

Figure S2. The dependence of pH for esterase activity on BnzGlcA. Activity profiles for (A) *As*CE15, (B) *Pd*CE15, (C) *Bi*CE15, (D) *Pv*CE15, and (E) *Pi*CE15A. Mean values of relative activity from duplicate measurements are plotted with standard error of the mean with the maximal activity taken as 100%.

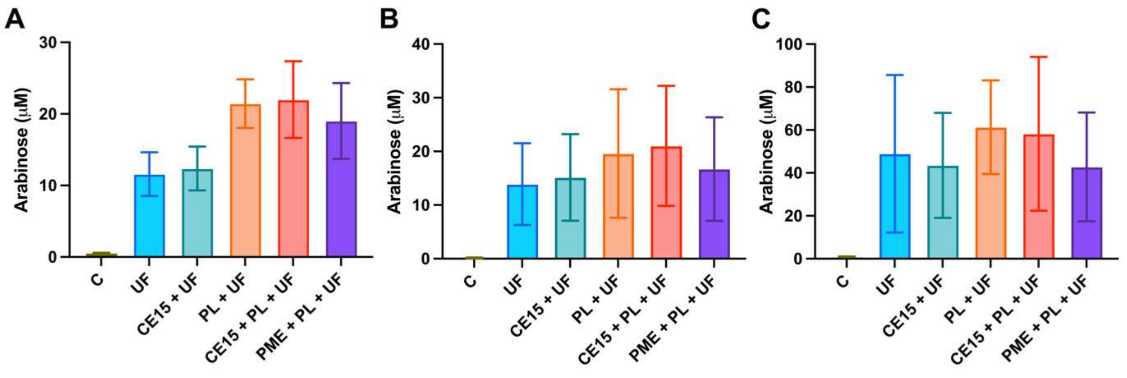

Figure S3. Effect of *Pv*CE15 on arabinose released during a 1-hour saccharification of different pectin rich biomasses. Biomass materials of (A) carrot pomace, (B) potato peels, and (C) orange peels were milled, washed, and resuspended into assays of 5 mg/mL and incubated for 1 hour at 30 °C with either UltraFlo (UF), *Pv*CE15 (CE15), pectate lyase (PL), or pectin methyl esterase (PME). Released monosaccharides from assays (N=4) were quantified by ion chromatography (HPAEC-PAD). The addition of *Pv*CE15 to either UltraFlo or UltraFlo supplemented with the pectate lyase did not lead to significant increases in released arabinose.

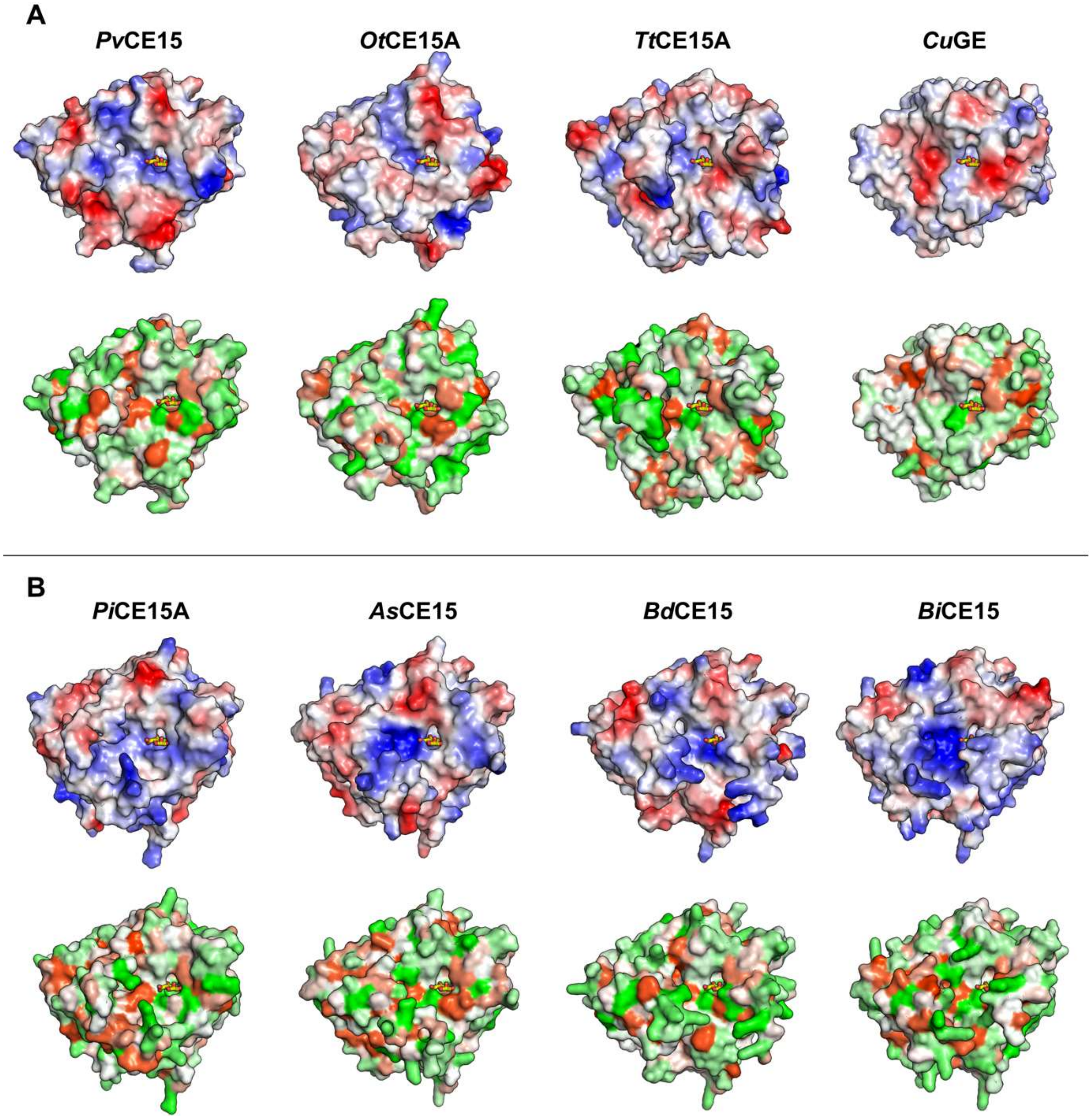

Figure S4. Comparison of predicted surface properties of CE15 structures. (A) Experimentally determined structures of *Pv*CE15, *Ot*CE15A (PDB accession: 6syr), *Tt*CE15A (PDB accession: 6hsw), and *Cu*GE (PDB accession: 6ru2), shown with (B) models of *Pi*CE15A, *As*CE15, *Pd*CE15, and *Bi*CE15 generated by AlphaFold in ColabFold (2). The surface representation of the proteins with coloring by (top) vacuum electrostatics defined Pymol with ranging from +100 to -100 with electronegative as red, electropositive as blue, and neutral as white. The surface representation of the proteins with coloring by (below) hydrophobicity with hydrophobic residues as orange, hydrophilic residues as green, and neutral as white. All structures are shown in the same orientation with the glucuronate observed in *Pv*CE15 (yellow sticks) placed in the other models by structural alignment.

Supplementary References

1. R. A. Engh, R. Huber, Accurate bond and angle parameters for X-ray protein structure refinement. *Acta Crystallographica Section A* **47**, 392-400 (1991).
2. M. Mirdita *et al.*, ColabFold: making protein folding accessible to all. *Nat Methods* **19**, 679-682 (2022).
